# Pharmacologic modulation of 5-fluorouracil by folinic acid and high-dose pyridoxine for treatment of patients with digestive tract carcinomas

**DOI:** 10.1038/s41598-021-92110-5

**Published:** 2021-06-16

**Authors:** David Machover, Wathek Almohamad, Vincent Castagné, Christophe Desterke, Léa Gomez, Yann Gaston-Mathé, Claude Boucheix, Emma Goldschmidt

**Affiliations:** 1 INSERM U935-UA09 and Institut de Cancérologie et d’Immunogénétique (ICIG), University Paris-Saclay, Hospital Paul-Brousse, 12, Avenue Paul-Vaillant-Couturier, 94800 Villejuif, France; 2 Department of Medical Oncology, Hospital Paul-Brousse, University Paris-Saclay, Assistance Publique-Hôpitaux de Paris (APHP), 94800 Villejuif, France; 3 Department of Pharmacy, University Paris-Saclay, Hospital Paul-Brousse, APHP, 94800 Villejuif, France; 4 Department of Biophysics and Nuclear Medicine, University Paris-Saclay, Hospital Kremlin-Bicêtre, APHP, 94270 Le Kremlin-Bicêtre, France; 5 YGM Consult SAS, 75015 Paris, France

**Keywords:** Cancer, Drug discovery, Oncology

## Abstract

Supplementation of cancer cells exposed to 5-fluorouracil (FUra) and folinic acid (FA) with high concentration pyridoxal 5′-phosphate, the cofactor of vitamin B6, potentiates the cytotoxicity of FUra in a synergistic interaction mode. We report a pilot study in 13 patients with previously untreated advanced carcinoma of the digestive tract to assess the impact of high-dose pyridoxine (PN) on the antitumor activity of regimens comprising FUra and FA. Five patients had colorectal adenocarcinoma (CRC); 5 had pancreas adenocarcinoma (PC); and 3 had squamous cell carcinoma of the esophagus (EC). Patients with CRC and with PC received oxaliplatin, irinotecan, FUra and FA, and patients with EC had paclitaxel, carboplatin, FUra and FA. PN iv from 1000 to 3000 mg/day preceded each administration of FA and FUra. Eleven patients responded. Two patients with CRC attained CRs and 3 had PRs with reduction rates ≥ 78%. Two patients with PC attained CRs, and 2 had PRs with reduction rates ≥ 79%. Responders experienced disappearance of most metastases. Of 3 patients with EC, 2 attained CRs. Median time to attain a response was 3 months. Unexpected toxicity did not occur. Results suggest that high-dose vitamin B6 enhances antitumor potency of regimens comprising FUra and FA.

## Introduction

Modulation of 5-fluorouracil (FUra) by folinic acid (5-formyl tetrahydropteroylglutamate; FA)^1,2^ is currently used in standard schemas for treatment of patients with colorectal, pancreas, and gastric carcinomas. This pharmacologic principle has been used to a lesser extent in patients with breast carcinoma and with head and neck squamous cell carcinoma, but not in all tumors covering the spectrum of antitumor activity of the fluoropyrimidine, which also includes ovarian, prostate, and bladder carcinomas^3^.

**Figure 1:**
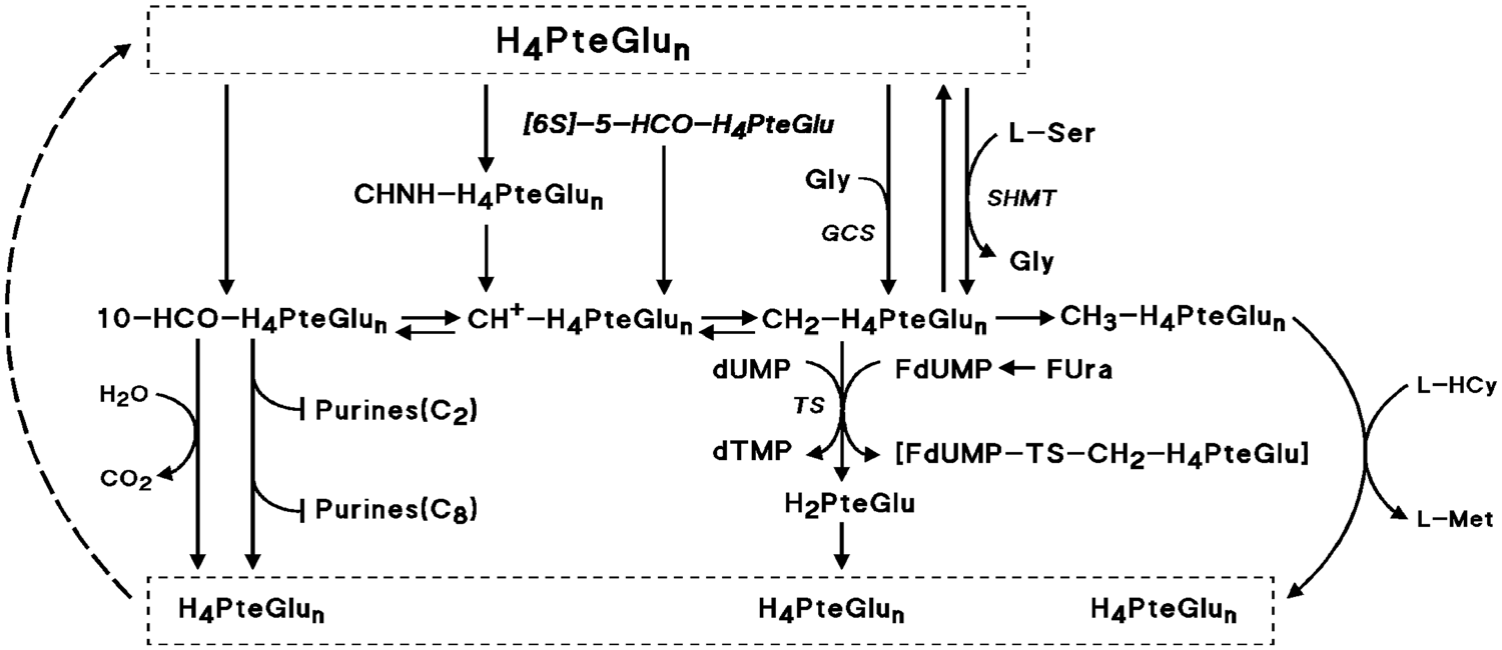
Selected Pathways of folates. Folates. H_2_PteGlu: 7,8-dihydrofolate; H_4_PteGlu: 5,6,7,8-tetrahydrofolate; CH_2_-H_4_PteGlu: 5,10-methylenetetrahydrofolate; CH_3_-H_4_PteGlu: 5-methyltetrahydrofolate; CH^+^-H_4_PteGlu: 5,10-methenyltetrahydrofolate; 10-HCO-H_4_PteGlu: 10-formyltetrahydrofolate; CHNH-H_4_PteGlu: 5-formiminotetrahydrofolate; [6S]-5-HCO-H_4_PteGlu: 5-formyltetrahydrofolate (FA; [6S]-leucovorin). Enzymes. TS, thymidylate synthase; SHMT, serine hydroxymethyltransferase (PLP-dependent enzyme, including the cytoplasmic SHMT1 and the mitochondrial SHMT2 isoforms); GCS, glycine cleavage system (mitochondrion) Other compounds and substances involved in TS inhibition. dUMP, deoxyuridylate; dTMP, thymidylate; L-HCy, L-homocysteine; L-Met, L-methionine; FUra, 5-fluorouracil; FdUMP, fluorodeoxyuridylate; [FdUMP-TS-CH_2_-H_4_PteGlu], the ternary complex resulting in inhibition of TS.

In the past decades, attempts at improvement of the anticancer effect of FUra and FA did not convincingly succeed. Probably, the cytotoxic activity of the combination has reached a limit that cannot be overcome by using other folates, higher doses of folates or changes in modalities of administration of the compounds. One explanation is that supplementation of cancer cells with reduced folates in any form or quantity results in small expansion of 5–10 methylene tetrahydropteroylglutamate (CH_2_-H_4_PteGlu) pools^4–11^ up to concentrations far below that required to increase the tightness of binding of fluorodeoxyuridine monophosphate (FdUMP), the active metabolite of FUra, to thymidylate synthase (TS) for maximum stability of the ternary complex [FdUMP-TS-CH_2_-H_4_PteGlu_n_] resulting in durable inhibition of the TS^12–15^ (Fig. 1). Stability of the complex was found to increase as CH_2_-H_4_PteGlu level is augmented over a wide concentration range up to levels higher than 500 µM^14,15^, these levels being much greater than that measured in cells exposed to high amounts of folates^4–11^. FdUMP-mediated inhibition of the TS prevents synthesis of thymidine triphosphate (dTTP) leading to deoxy nucleotide triphosphate (dNTP) pool imbalance, and results in accumulation of deoxy uridine triphosphate (dUTP) and fluorodeoxyuridine triphosphate (FdUTP), which lead to genomic DNA replication defects including DNA mismatch and altered replication fork progression eliciting DNA damage cell responses and, ultimately, cell death^16–19^.

Studies of serine hydroxymethyl transferase (SHMT), a ubiquitous pyridoxal 5′-phosphate (PLP)-dependent enzyme that catalyzes the transfer of the beta carbon (Cβ) of L-serine to tetra hydro pteroylglutamate (H_4_PteGlu) with formation of glycine and CH_2_-H_4_PteGlu, determined that the dissociation constant (K_d_) values for binding of apo SHMT isoforms to cofactor are much greater than intracellular PLP concentration levels. SMHT from various sources was found to bind to cofactor with K_d_ from 250 nM to as high as 27 µM^20–23^, while naturally occurring PLP levels in erythrocytes vary from 30 to 100 nmol/L of packed cells^24,25^, which indicates that SHMT activity should be sensitive to PLP concentration modifications. Studies reported changes of folate-mediated one-carbon metabolism through SHMT catalysis related to vitamin B6 availability in rat liver^26^, and in the MCF-7 human mammary carcinoma cell line^21^. In the latter study, addition of PLP resulted in great increase of SHMT intracellular activity^21^.

From these data we assumed that, in tumors, naturally occurring PLP levels are too small to allow intracellular SHMT-dependent conversion of H_4_PteGlu into CH_2_-H_4_PteGlu in amounts required to improve inhibition of TS by FdUMP by stabilizing the ternary complex^27^. To test for variations of SHMT activity resulting from PLP level changes in tumor cells, we conducted experiments in the human colon carcinoma HT29, and HCT116 cell lines, and in the murine leukemia L1210 cell line in vitro to investigate for interactions between FUra, FA, and PLP on cell growth^27^. Supplementation of cancer cells exposed to FUra with high concentrations of PLP and FA strongly potentiated the cytotoxic activity of FUra in the three cell lines and resulted in synergistic interaction in HT29 and in L1210 cells, while summation was found in HCT116 cells. Experiments in mice have shown that erythrocyte levels of PLP after parenteral administration of high dose B6 vitamer in the form of pyridoxamine (PM), or pyridoxine (PN), rise to concentrations within the range of K_d_ values of SHMT binding to cofactor, suggesting that modulation of FUra by vitamin B6 could be achieved in vivo^27^. These studies also demonstrated that newly synthesized PLP was rapidly cleared from cells; levels reached baseline concentrations by 12 h after injection, with no cumulative effect when administrations were repeated at 12-h interval. Rapid decline of intracellular PLP levels after vitamin B6 administration has been reported in man^24,25^. From these data, we thought that administration of high-dose B6 vitamer to patients treated with FUra and FA would increase intracellular PLP levels within tumors, leading to augmentation of CH_2_-H_4_PteGlu synthesis resulting in long-term inhibition of TS and enhanced antitumor effect.

We report herein a translational pilot study in previously untreated patients with advanced-stage carcinomas of the digestive tract whose standard treatment included a combination of FUra and FA, consisting in addition of pyridoxine in high dose to these regimens. Vitamin B6 in high doses given for treatment of various conditions was reported to be safe in man^28,29^. However, pyridoxine in very high doses for long periods of time has caused neurologic toxicity in the form of sensory peripheral neuropathy^28^. From these data, we thought that vitamin B6 administered in short-time courses followed by drug-free intervals in doses far below that reported to be toxic in man, was not likely to expose patients to increased risk of neuropathy. However, particular attention was put on neurologic signs and symptoms during the course of the study.

We used a casuistic analytic approach because of the limited number of patients included in the present pilot study. We describe data from 13 patients who were entered from May 2014 to October 2020.

## Materials and methods

The study was approved by the Medical Oncology Department board in Paul-Brousse Hospital, Assistance Publique-Hôpitaux de Paris, and University Paris-Saclay. It was conducted in accordance with the basic principles of the Declaration of Helsinki. All the patients were informed of the rationale, potential benefits and risks of the treatment. Written informed consent to study participation was obtained from all patients.

### Patients

Patients with three types of carcinoma of the digestive tract in advanced stages carrying poor prognostic features who had not received prior treatment were entered, including patients with poor performance status (PS) scores whose nutritional and vital function conditions were sufficiently preserved to allow chemotherapy being administered cautiously in safe conditions. Owing to the great extent of tumor at presentation and poor PS scores in most, patients could not be selected neither for resection surgery nor for any investigational therapy available.

Thirteen patients were included. Five patients aged 37–65 years old had advanced colorectal adenocarcinoma with regional invasion and great numbers of metastases (Table 1). Of these, two had adenocarcinoma with wild type (WT) Ras, and three patients had tumors that carried activating K-Ras mutations. One patient had initial resection of the primary tumor and four did not. Five patients aged 50 to 71 years old had locally advanced unresectable pancreas adenocarcinoma of which 3 had numerous distant metastases. Of these, 4 had carcinoma of the head of pancreas complicated with obstructive cholestasis requiring implantation of biliary stents, and 1 had carcinoma originating in the tail. Three patients aged 62 to 77 years old had squamous cell carcinoma of the upper third or in limit of the upper and mid thirds of the esophagus that could not be resected; one had tumor originating in the esophagus comprised within the radiation field for prior treatment of breast cancer, one had poorly differentiated squamous cell carcinoma with high proliferative rate, and the other patient had lymph node involvement in mediastinum (Table 1). Eastern Cooperative Oncology Group (ECOG) PS scores at presentation were 1 in one patient, 2 in 4 patients, and 3–4 in 8 patients (Table 1).

**Table 1:** Characteristics of patients treated with regimens including 5-fluorouracil, folinic acid, and pyridoxine in tandem.

Patient	Sex (Age)^1^	Organ of origin. Initial surgery	Type of tumor	Estimated tumor extent at presentation (site of tumor, and approximate number of metastases by site)	ECOG PS
1	M (65)	Caecum. Hemicolectomy	Poorly differentiated adenocarcinoma; Ras-WT	Peritoneum (disseminated)^2^	2
1-R	M (68)	Explorative laparoscopy	Poorly differentiated adenocarcinoma; Ras-WT	Peritoneum (disseminated)^3^	3
2	F (37)	Right colon. No resection	Adenocarcinoma K-Ras mutated (c.35G>A; p.G12D)	Primary tumor.^3^ Liver (> 100)	4
3	F (60)	Rectum. Colostomy	Poorly differentiated adenocarcinoma K-Ras mutated (c.35G>A; p.G12D)	Primary tumor.^3^ Liver (2). Lung (> 100), pleura. Peritoneum (mesorectum, pelvis). Bone (sacrum). Nodes (abdomen, thorax)	4
4	F (61)	Right colon. No resection	Poorly differentiated adenocarcinoma K-Ras mutated (c.34G>T; p.G12C)	Primary tumor.^3^ Lung (> 40). Peritoneum. Nodes (abdomen; pelvis)	2
5	M (49)	Caecum. No resection	Adenocarcinoma; Ras-WT	Primary tumor.^3^ Liver (> 150). Peritoneum. Nodes (abdomen; pelvis)	3
6	M (71)	Pancreas, head-body. No resection	Well differentiated adenocarcinoma	Primary tumor.^4^ Invasion of portal vein, celiac artery, and duodenum	3
7	F (69)	Pancreas, head. No resection	Carcinoma	Primary tumor.^4^ Invasion of celiac artery. Lung (11). Nodes (abdomen; thorax)	3
8	M (50)	Pancreas, tail. No resection	Adenocarcinoma	Primary tumor. Invasion of celiac artery. Liver (disseminated)^2^	4
9	F (61)	Pancreas, head. No resection	Adenocarcinoma	Primary tumor.^4^ Invasion of celiac artery. Liver (9). Lung (miliary).^2^ Nodes (abdomen)	3
10	M (67)	Pancreas, head. No resection	Adenocarcinoma	Primary tumor.^4^ Invasion of portal vein, celiac artery	3
11	F (74)	Esophagus, upper third within field of prior radiotherapy. No resection	Well differentiated squamous cell carcinoma	Primary tumor; EUS T3N1	1
11-R	F (77)	Esophagus, upper third within field of prior radiotherapy. No resection	Poorly differentiated squamous cell carcinoma	Primary tumor; EUS T2N0	1
12	M (62)	Esophagus, limit upper-mid third. No resection	Poorly differentiated squamous cell carcinoma	Primary tumor; EUS T2N1	2
13	F (77)	Esophagus, upper-mid third. No resection	Poorly differentiated squamous-cell carcinoma	Primary tumor. Nodes (mediastinum)	2

^1^Age at start of treatment with FUra, FA and B6 in tandem.

^2^Presence of countless tumor foci.

^3^Patient with partial bowel obstruction.

^4^Patient with obstructive cholestasis.

EUS, endoscopic ultrasonography. R, relapse.

### Treatment

Patients received the standard therapeutic regimens comprising a combination of FUra and FA that were indicated for treatment of their disease, supplemented with pyridoxine in high doses accompanying each administration of FA plus FUra (Table 2).

**Table 2:** Results of therapy in patients treated with regimens comprising 5-fluorouracil, folinic acid, and pyridoxine in tandem.

Patient	Regimen comprising FUra, FA and PN in tandem^1^	Median PN dose (range)^2^ in mg/day [total no. of courses administered]	Antitumor activity			Tumor markers Start/After treatment CEA (ng/ml) CA19-9 (U/ml)	Time to response (Mo.)^6^	PFS (Mo.) [Mo. off therapy]
			Clinical (RECIST)^3^	Metabolic (PERCIST)^4^	Pathologic^5^			
1	Folfirinox + cetuximab	1500 (1000–2000) [40]	− 100	NA	–	–	2.9	44 [25.1]
1-R	Folfirinox + cetuximab	3000 (2000–3000) [19 +]	− 48	NA	–	–	2.6	9.4 +
2	Folfirinox	3000 (2000–3000) [13]	− 87^7^	− 100	ypT3N1bM1a	CEA: 6667/4.1	3	8.6
3	Folfirinox	3000 (2000–3000) [12]	− 86^7,8,9^	NA	–	CEA: 151/5.5 CA19-9: 2376/15.1	2.7	10.8
4	Folfirinox	3000 (2000–3000) [23+]	− 81^8^	− 100	ypT0N0M0	–	3.8	13.5 +
5	Folfirinox + cetuximab	1000 (1000) [16]	− 78^7^	− 69	ypT4bN2aM1c	CEA: 12/3.5 CA19-9: 2073/18.3	3.1	9.2
6	Folfirinox	1000 (1000–3000) [22]	− 100	NA	ypT3N0M0	CA19-9: 9728/1.8	4.5	23.2 [11.4]
7	Folfirinox	1000 (1000–3000) [37]	− 100	− 100	–	CA19-9: 9755/24.7	4.8	26.7
8	Folfirinox	3000 (3000) [6]	− 86^7^	NA	–	CA19-9: 9756/2646	2.2	5.7
9	Folfirinox	1000 (1000–1500) [29]	− 79^7,8^	NA	–	–	3.8	14.3
10	Folfirinox	1500 (1000–3000) [10]	− 16	0	–	–	–	6.5
11	TXL-CBDCA-FUra-FA	3000 (2000–3000) [8]	− 100	− 100	T0N0^10^	–	1.4	30.2 [24.3]
11-R	TXL-CBDCA-FUra-FA	2000 (2000–2500) [5]	− 100	− 100	T0N0^10^	–	2	33.1 + [30.3 +]
12	TXL-CBDCA-FUra-FA	2000 (2000) [6]	− 100	− 100	ypT0N0M0	–	1.1	64.3 + [58.1 +]
13	TXL-CBDCA-FUra-FA	1500 (1000–1500) [6]	− 29	− 18	–	–	–	5.6

^1^In patients treated with Folfirinox who required suspension of the L-OHP due to peripheral neuropathy, chemotherapy was pursued as Folfiri.

^2^Range represents the intra-patient dose escalation of PN preceding each injection of FUra and FA given for a number of days defined by the chemotherapy regimen used.

^3^Percent variation in sum of greatest diameters was assessed by CT scan, MRI, and endoscopic ultrasonography (EUS) imaging when required. For patients who attained a response accompanied by disappearance of most metastases, RECIST values were calculated by size comparison of persisting tumors at the time of assessment with these same tumors before treatment.

^4^Percent variation of peak standard ^18^FDG uptake value normalized by lean body mass (SUL_peak_) of targets as measured by Positron Emission Tomography (PET) scan.

^5^Pathologic response (AJCC) was assessed by colectomy and hepatectomy (Patients 2, and 5), colectomy and pulmonary resection (Patient 4), pancreaticoduodenectomy (Patient 6), endoscopic ultrasonography-guided biopsy (Patient 11), and esophagectomy (Patient 12) ^6^Time to attain antitumor response, *i.e.*, a reduction in sum of diameters by ≥ 30%. Response was accompanied by disappearance of most liver,^7^ lung^8^ and/or bone^9^ metastases present before treatment together with major reduction of all perceptible tumor foci.

^10^Absence of residual tumor in EUS-guided esophageal and transmural node biopsies. R, second line therapy after relapse. NA, not assessable.

Patients with colorectal carcinoma and with pancreas adenocarcinoma had induction treatment using the regimen said Folfirinox consisting in two-day courses repeated every 14 days of combined oxaliplatin (L-OHP; 85 mg/m^2^, Day 1), irinotecan (CPT11; 180 mg/m^2^, Day1), folinic acid (FA; [6R,S]-5-formyl tetrahydropteroylglutamate; [6R,S]-5-HCO-H_4_PteGlu; 200 mg/m^2^/day, Days 1 and 2), and 5-fluorouracil (FUra; 1000 mg/m^2^day, Days 1 and 2) distributed in one rapid iv injection (400 mg/m^2^/day), and one iv infusion during 22 h (600 mg/m^2^/day). We used the Folfirinox regimen described above instead of slightly different simplified versions^30^, in order to insure two consecutive days of exposure to the modulators FA and PN, owing to the rapid plasma and cellular clearance of their metabolites whose intracellular pool expansion forms the rationale of the present study^11,24,25,27,31^. Patients with Ras-WT colorectal carcinoma received the anti EGF-R chimeric monoclonal antibody cetuximab in addition to chemotherapy every 14 days. One complete responder with CRC, and one with PC who had further tumor progression received the same regimen as initially for treatment of relapse (Tables 1, 2, Fig. 3). Oxaliplatin was suspended when Grade 2 symptoms of sensory peripheral neuropathy (i.e., moderate hypoesthesia, paresthesia and/or dysesthesia limiting activities of daily living) lasting ≥ 1 week from the previous L-OHP injection, and/or the Lhermitte’s sign were first recorded, and then treatment was pursued with no supplementary change (*i.e.*, as said Folfiri regimen combined with pyridoxine). Patients with squamous-cell carcinoma of the esophagus had four-day courses repeated every 21 days of combined paclitaxel (TXL; 175 mg/m^2^, Day 1), carboplatin (CBDCA; AUC = 5, Day 1), FA (200 mg/m^2^/day, Days 1–4), and FUra (400 mg/m^2^/day, Days 1–4) iv in 2 h. The present schema was adapted from regimens previously described for treatment of patients with esophageal and head and neck carcinoma, to be administered in courses of short duration^32,33^. One complete responder with carcinoma of the esophagus who had further tumor progression received the same regimen as initially for treatment of relapse (Table 1).

Vitamin B6 is the generic name that encompasses six interconvertible compounds (i.e., B6 vitamers), namely pyridoxine (PN); pyridoxamine (PM); pyridoxal (PL); and their respective 5′ phosphorylated forms, pyridoxine 5′-phosphate, pyridoxamine 5′-phosphate, and the cofactor pyridoxal 5′-phosphate (PLP)^24,25,27^. Pyridoxine hydrochloride, the only available marketed parenteral B6 vitamer for clinical use (in 250 mg vials) was injected iv in 30’ preceding each injection of FA and FUra for a number of days defined by the schedule of the regimen used. Based on the pharmacokinetic data obtained in mice^27^, using approximate factors for converting doses in man from mouse data^34^, the daily dose of pyridoxine was augmented in patients over the duration of the present study from 1000 mg/day to a maximum of 3000 mg/day. The latter corresponds approximately to the highest dose of PM and PN explored in mice^27^; in these animals, it resulted in rise of intracellular concentrations of PLP to peak levels within the range of most reported K_d_ values for binding of PLP to apo SHMT, the requirement that supports the rationale underlying the present clinical study^20–23,27^.

The first starting dose of PN accompanying each administration of FUra and FA was 1000 mg/day. Then, we practiced stepwise intra patient dose escalation of pyridoxine by increments of 500 to 1000 mg/day in subsequent courses. In absence of any form of toxicity seeming attributable to the PN recorded in prior patients, the starting daily dose of PN in next patients was increased to 2000 mg/day, and then to a maximum of 3000 mg/day (Table 2). Treatment courses were repeated in each patient until antitumor response of the maximum degree was attained, and then patients received an a priori undefined number of courses in a personalized way according to patient’s condition and decisions from referring oncologists and clinical meetings.

## Results

Antitumor response was assessed by studying the variation of the sum of diameters of measurable tumors according to Response Evaluation Criteria in Solid Tumors (RECIST), and that of peak standard ^18^ FDG uptake value normalized by lean body mass (SUL_peak_) of targets as measured by Positron Emission Tomography (PET) scan according to PET Response Evaluation Criteria in Solid Tumors (PERCIST), together with periodic clinical examination and measurement of plasma tumor markers. Assessment of pathologic response was obtained in several complete responders and in patients who had achieved partial responses of high magnitude who were subjected to locoregional resection with eradication intent.

Of 13 patients included, 11 responded to therapy and two did not. Induction treatment resulted in antitumor responses of early onset and great magnitude.

### Patients with colorectal adenocarcinoma

**Figure 2:**
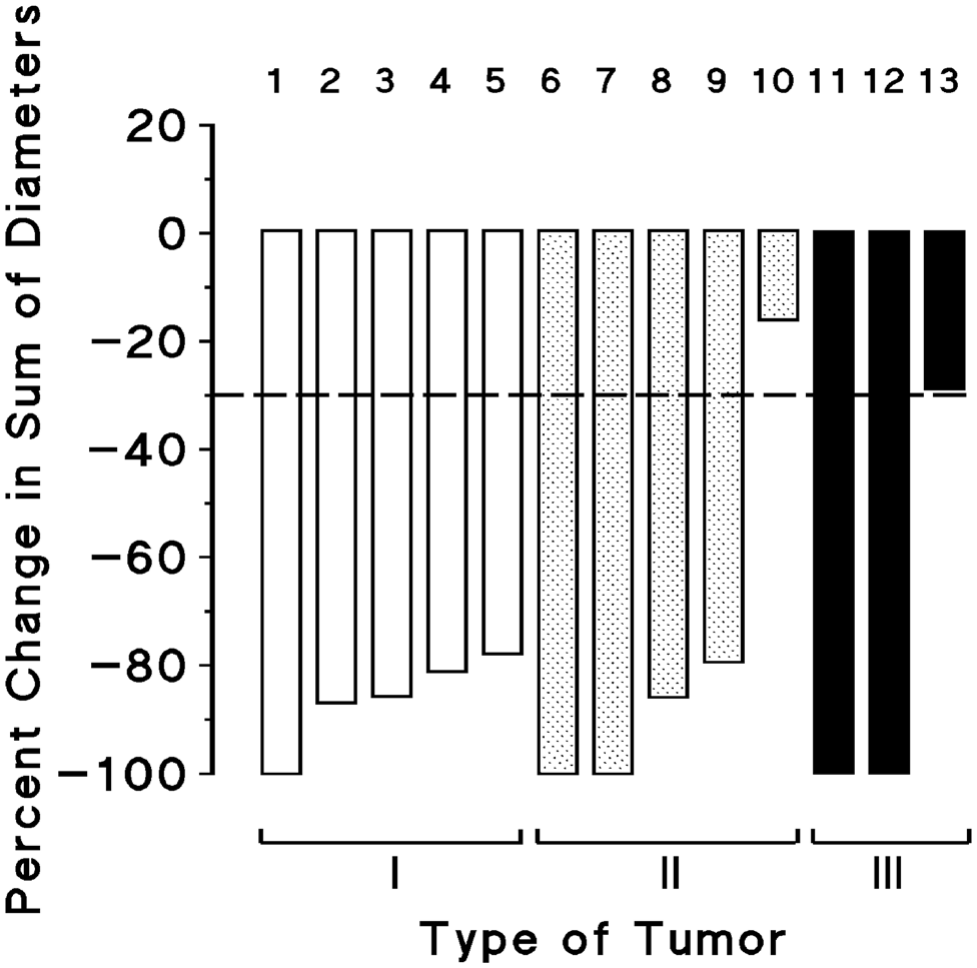
Clinical antitumor response in 13 patients treated with regimens comprising 5-fluorouracil, folinic acid, and high-dose pyridoxine in tandem. Types of tumor included colorectal adenocarcinoma (I), pancreas adenocarcinoma (II), and squamous cell carcinoma of the esophagus (III). Clinical assessment comprised CT scan, MRI, and/or endoscopic ultrasonography (EUS) imaging when required. In patients who had great numbers of targets who attained a response accompanied by disappearance of most metastases (patients 2, 3, 4, 5, 8, and 9), calculations of percent reduction in sum of greatest diameters (RECIST) were done by size comparison of remaining images at the time of assessment with these same tumor images present before treatment. The discontinuous line at − 30%, represents the limit between no change and antitumor response One complete responder with colorectal carcinoma (Patient 4) had reduction in sum of diameters by 81% as measured by CT scan owing to persisting nodular scars in lungs, together with metabolic CR, and pathologic CR (Table 2). The other complete responder with colorectal carcinoma (Patient 1) and one with esophageal carcinoma (Patient 11) had subsequent relapse. Then, patients were subjected to the same treatment as for first induction and attained a PR (percent reduction, 48%), and a CR, respectively; these second line responses are not represented in the Figure (see Table 2, Fig. 3).

**Figure 3:**
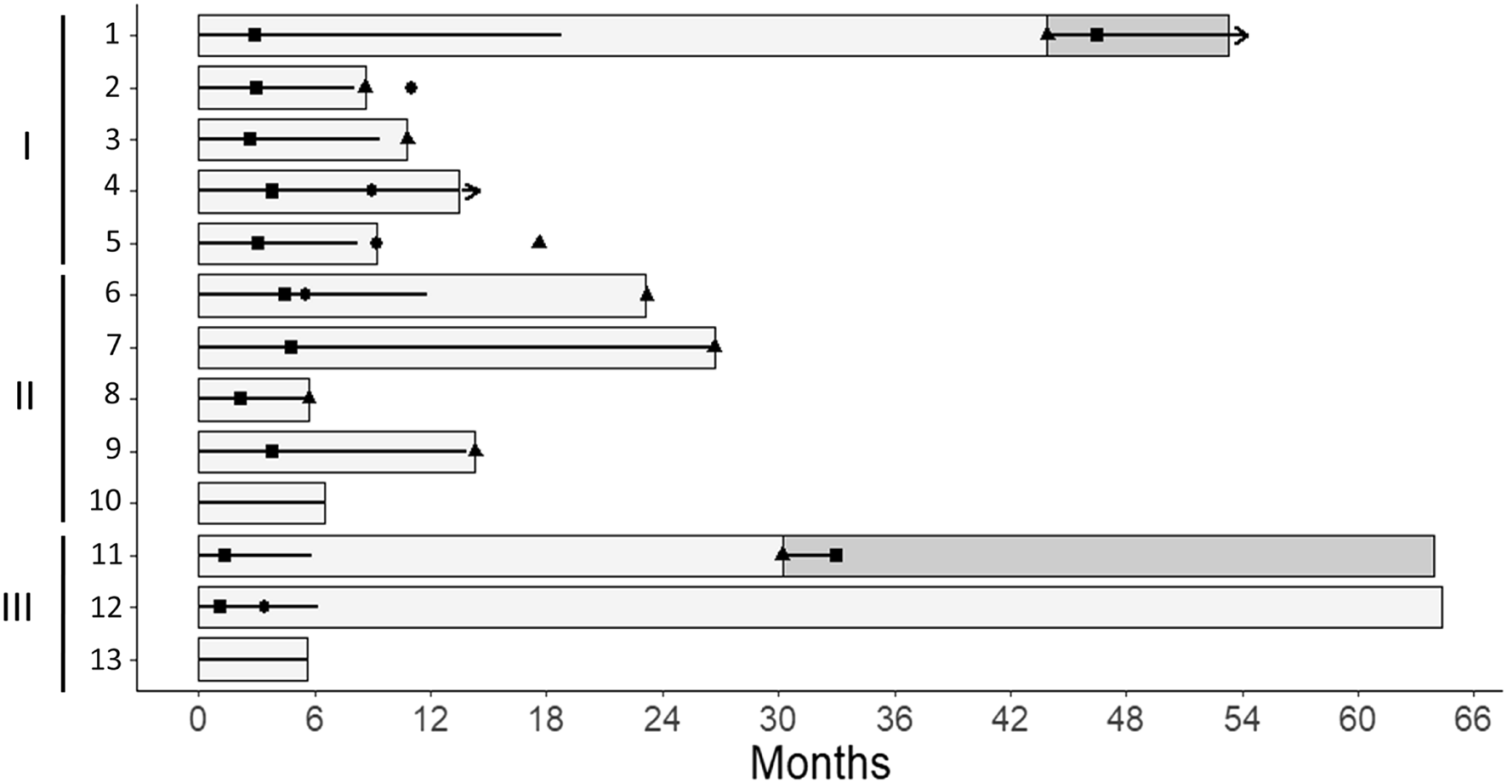
Chronological sequence of events in 13 patients treated with regimens comprising 5-fluorouracil, folinic acid, and high-dose pyridoxine in tandem. Types of tumor included colorectal adenocarcinoma (I), pancreas adenocarcinoma (II), and squamous cell carcinoma of the esophagus (III). Patients are numbered in the same order as in Tables 1, and 2, and in Fig. 2. Bars represent PFS times (light grey, and dark grey bars indicate 1st, and 2nd PFS times, respectively). Bold black lines within bars represent duration of treatment, and arrows indicate ongoing treatment. Solid squares indicate the time when response to therapy was recorded. Solid circles represent the time when surgery with eradication intent was performed. Solid triangles indicate the time when tumor progression was recorded in previous responders to therapy.

Of the 5 patients, two attained a CR and 3 had PRs with great tumor reduction rates (percent reduction in sum of longest diameters were 78, 86, and 87%), accompanied with disappearance of most metastases that were present before treatment (Table 2, Fig. 2). Progression-free survival (PFS) time of one complete responder was 44 months, a relapse occurring after 25 months off therapy. The patient was then subjected to the same treatment as for first induction and attained a PR (percent reduction in diameter was 48%) of 9.4 + months. The second complete responder had reduction in sum of diameters by 81% as measured by CT scan owing to persisting nodular scars in lungs, together with metabolic CR, and pathologic CR assessed by colectomy and resection of lung nodules that contained scar tissue without residual tumor; PFS time was 13.5 + months. The three partial responders had tumor growth despite continued treatment after PFS times of 8.6, 9.2, and 10.8 months (Table 2, Fig. 3). Plasma tumor markers were normalized in the three patients who had high marker levels before initiation of treatment (Table 2). Times to attain a response ranged from 2.7 to 3.8 months.

### Patients with pancreas adenocarcinoma

Of the 5 patients, two attained a CR, two had a PR with great tumor reduction rates (percent reduction in sum of diameters were 79, and 86%) and one patient had no change (Table 2, Fig. 2). One complete responder had pancreaticoduodenectomy resulting in removal of residual tumor; PFS time was 23.2 months, including 11.4 months off therapy. At relapse, the patient was subjected to the same treatment as for induction but failed to respond. The second complete responder had tumor growth despite continued treatment after PFS time of 26.7 months. One partial responder had tumor reduction by 79%, together with disappearance of most miliary metastatic nodules in lungs; tumor growth occurred under treatment after 14.3-month PFS time. The second partial responder had tumor reduction by 86% together with disappearance of countless liver metastases; tumor growth occurred under treatment after PFS time of 5.7 months. Times to attain a response ranged from 2.2 to 4.8 months (Table 2, Fig. 3). Plasma tumor markers were normalized in the two complete responders. One partial responder had decrease of markers by approximately 75% from baseline value (Table 2).

### Patients with squamous-cell carcinoma of the esophagus

Of the 3 patients, two attained histologically proven CRs, and one patient had no change. One complete responder with tumor occurring within the field of prior radiotherapy administered for breast cancer had PFS time of 30.2 months and relapsed after 24.3 months off therapy. Then, the patient was subjected to the same treatment as for first induction and attained second histologically proven CR with PFS time of 33.1 + months, including 30.3 + months off therapy. The second complete responder had esophagectomy that did not find any residual tumor in resected tissue; PFS time was 64.3 + months, including 58.1 + months off therapy. Times to attain a response were 1.1, and 1.4 months (Table 2, Figs. 2, 3).

Selected iconography for the 11 responders to therapy is shown in Table 3.

**Table 3:**
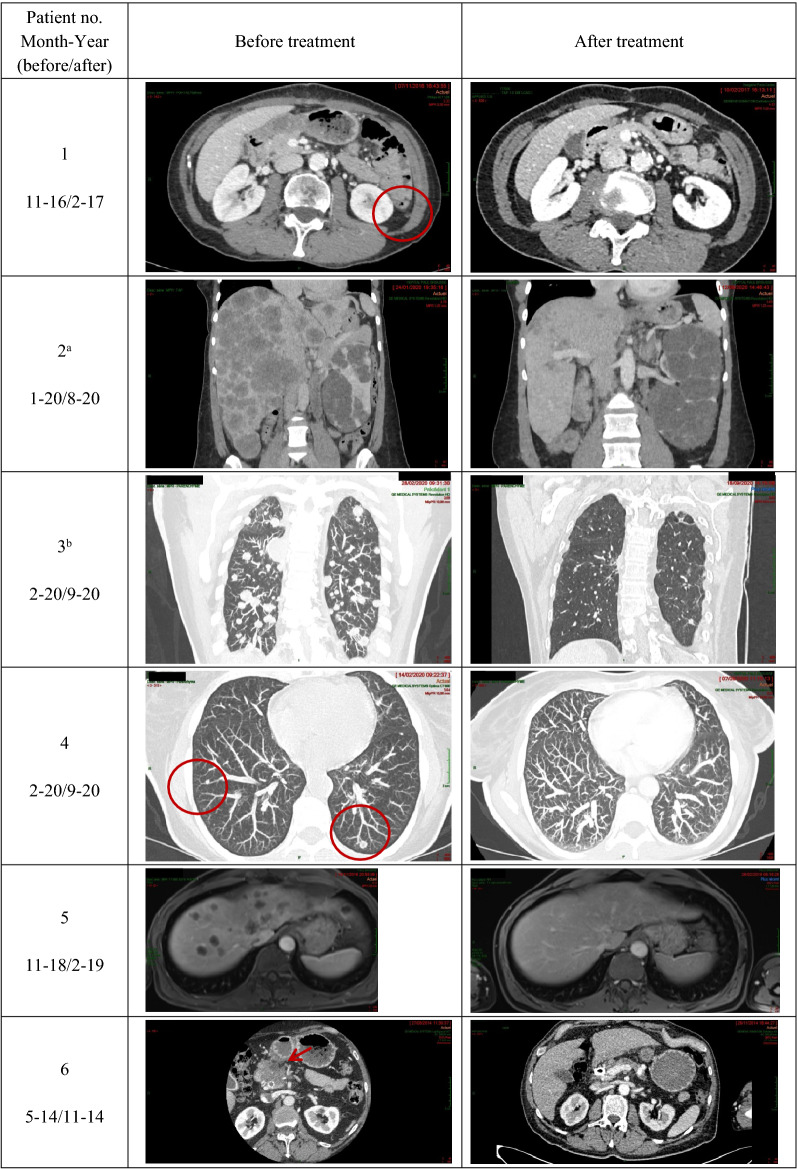

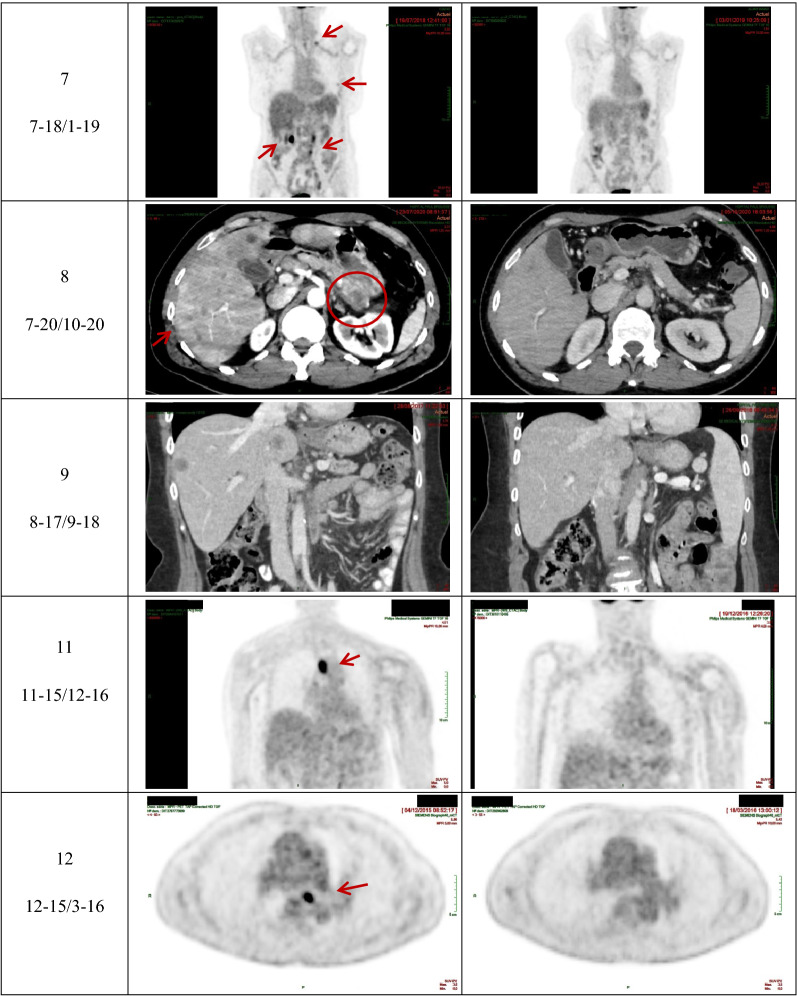
Selected iconography of the 11 responders to regimens comprising 5-fluorouracil, folinic acid, and pyridoxine in tandem. Patients are numbered in the same order as in Tables 1, and 2, Fig. 2, and Fig. 3

^**a**^Presence of polycystic kidneys.

^**b**^Presence of CoViD-19 pneumonia-related images in CT scan after treatment.

Assessment of toxicity before initiation of each cycle of therapy did not record neither any form of unusual toxicity nor toxic effect of greater magnitude than that expected with each particular regimen used^31,35^. Interruption of the L-OHP due to sensory peripheral neuropathy occurred in the 10 patients who were treated with the Folfirinox regimen, at cumulative doses of L-OHP ranging from 442 to 1044 mg/m^2^ (median, 690 mg/m^2^). Patients, whose chemotherapy was pursued as Folfiri plus pyridoxine, had further progressive decrease of neurologic symptoms. No signs of peripheral neuropathy were observed in the 3 patients with carcinoma of the esophagus. Except for interruption of oxaliplatin due to dose cumulative neuropathy, no dose reductions of any cytostatic agent or increasing intervals between courses were required.

## Discussion

Antitumor responses of great magnitude were attained by 11 of 13 previously untreated patients with unresectable carcinoma of the digestive tract, of which most had numerous metastases. Complete responses of long duration and partial responses with high tumor reduction rates were achieved by patients with the three types of tumors treated in the study. Response to therapy was rapidly attained; the number of courses required to attain a response (either a PR or a CR) ranged from 2 to 10 (median, 5 courses).

First-line Folfirinox has proven efficacy in patients with advanced colorectal and pancreas adenocarcinoma. Antitumor responses have been reported in approximately 30% of patients with pancreas carcinoma^30^, and in rates as high as 75% of patients with colorectal carcinoma^36–38^. In patients with squamous cell carcinoma of the esophagus, FUra plus platinum analog-containing regimens have been reported to produce response rates as high as 60%^32^. However, complete responses as well as partial responses of great magnitude are infrequent events in patients with these neoplasms.

Response rates from the present study cannot be compared to data from series with higher numbers of patients. However, the great magnitude of antitumor responses of long duration reported herein, which were rapidly attained by patients who carried in most cases high tumor burden, suggest that addition of vitamin B6 in high dose may strongly enhance the antitumor activity of standard chemotherapy combination regimens comprising FUra and FA.

Magnitude of the antitumor response and how quickly responses are attained have been reported as marks of antitumor potency in solid tumors. Improvement of long-term prognosis related to early tumor shrinkage, and to depth of antitumor response under induction cytostatic treatment was demonstrated from studies of patients with advanced stage colorectal carcinoma^38,39^. Progression-free survival and survival times were greater in patients who achieved early tumor shrinkage and were increased with increasing magnitude of response. In one study^38^, patients whose tumor reduction rate was greater than 70% experienced the best long-term outcome. However, magnitude of responses of such high levels are attained by less than 25 percent of patients with advanced colorectal carcinoma subjected to induction chemotherapy with FUra, folinic acid and oxaliplatin or with Folfirinox^36–38^, which emphasizes the need for powerful new strategies applicable to patients with this neoplasm in need of chemotherapy.

The present preliminary study does not enable correlating the magnitude of antitumor responses nor the rapidity to attain a response with the median daily dose of pyridoxine preceding each injection of FUra and FA received by each patient during the time of treatment (Table 2). Larger trials including dose finding studies are necessary to explore this issue.

Assessment of toxicity due to treatment could not discover any acute form of toxicity greater than that expected with each particular regimen, nor any unexpected toxic effect. In particular, the use of high cumulated doses of pyridoxine was not accompanied with greater levels, or higher grades, of sensory peripheral neuropathy than that expected with the use of the oxaliplatin as part of the Folfirinox regimen. In patients who had oxaliplatin, the cumulative amount received by patients when dose-limiting sensory peripheral neuropathy occurred was within the range of that reported in earlier single drug phase II studies^35^. Similarly, comparisons with data obtained in earlier phase II studies of FUra combined with the active (6S)-stereoisomer of folinic acid, did not reveal greater levels of FUra-related type of toxicity including mucositis, lacrimation, dermatitis and diarrhea than that previously reported^31^.

The remarkable favorable results observed in the present pilot study may represent the difference with that reported elsewhere with combination regimens comprising FUra and FA administered in their standard form. Owing to the limited number of patients entered in the present pilot study, further investigations are needed to demonstrate our findings. Exploring potentiation of FUra by FA and high dose B6 vitamer in tandem requires a first step of phase II trials in patients with potentially FUra-sensitive carcinomas. Vitamin B6 pharmacokinetic and dose-finding studies with emphasis on intracellular PLP levels, should accompany these trials to optimize the modulation of the fluoropyrimidines in accordance with experimental data.

## References

[1] Machover D (1982). Treatment of advanced colorectal and gastric adenocarcinomas with 5-FU combined with high-dose folinic acid: A pilot study. Cancer Treat Rep..

[2] Piedbois P (1992). For the Advanced Colorectal Cancer Meta-Analysis Project. Modulation of 5-fluorouracil by leucovorin in patients with advanced colorectal cancer: Evidence in terms of response rate. J. Clin. Oncol..

[3] Livingstone RB, Carter SK, Livingston RB, Carter SK (1970). 5-Fluorouracil. Single Agents in Cancer Chemotherapy.

[4] Houghton JA (1990). Influence of dose of [6RS] leucovorin on reduced folate pools and 5-fluorouracil-mediated thymidylate synthase inhibition in human colon adenocarcinoma xenografts. Cancer Res..

[5] Voeller D, Allegra CJ (1994). Intracellular metabolism of 5-methyltetrahydrofolate and 5-formyltetrahydrofolate in a human breast-cancer cell line. Cancer Chemother. Pharmacol..

[6] Wright JE (1989). Selective expansion of 5,10-methylenetetrahydrofolate pools and modulation of 5-fluorouracil antitumor activity by leucovorin in vivo. Cancer Res..

[7] Romanini A (1991). Role of folylpolyglutamates in biochemical modulation of fluoropyrimidines by leucovorin. Cancer Res..

[8] Zhang Z-G, Rustum YM (1991). Effects of diastereoisomers of 5-formyltetrahydrofolate on cellular growth, sensitivity to 5-fluoro-2′-deoxyuridine, and methylenetetrahydrofolate polyglutamate levels in HCT-8 cells. Cancer Res..

[9] Boarman DM, Allegra CJ (1992). Intracellular metabolism of 5-formyl tetrahydrofolate in human breast and colon cell lines. Cancer Res..

[10] Machover D (2001). Cytotoxic synergism of methioninase in combination with 5-fluorouracil and folinic acid. Biochem. Pharmacol..

[11] Houghton JA (1990). Relationship between dose rate of [6*RS*]leucovorin administration, plasma concentrations of reduced folates, and pools of 5,10-methylenetetrahydrofolates and tetrahydrofolates in human colon adenocarcinoma xenografts. Cancer Res..

[12] Santi DV, McHenry CS, Sommer H (1974). Mechanism of interaction of thymidylate synthetase with 5-fluorodeoxyuridylate. Biochemistry.

[13] Ullman B, Lee M, Martin DW, Santi DV (1978). Cytotoxicity of 5-fluoro-2′-deoxyuridine: Requirement for reduced folate cofactors and antagonism by methotrexate. Proc. Natl. Acad. Sci. USA.

[14] Danenberg PV, Danenberg KD (1978). Effect of 5,10-methylenetetrahydrofolate on the dissociation of 5-fluoro-2′-deoxyuridylate from thymidylate synthetase: Evidence for an ordered mechanism. Biochemistry.

[15] Lockshin A, Danenberg PV (1981). Biochemical factors affecting the tightness of 5-fluorodeoxyuridylate binding to human thymidylate synthetase. Biochem. Pharmacol..

[16] Wyatt MD, Wilson DM (2009). Participation of DNA repair in the response to 5-fluorouracil. Cell Mol. Life Sci..

[17] Mani C, Pai S, Papke CM, Palle K, Gmeiner WH (2018). Thymineless death by the fluoropyrimidine polymer F10 involves replication fork collapse and is enhanced by Chk1 inhibition. Neoplasia.

[18] Cortez D (2015). Preventing replication fork collapse to maintain genome integrity. DNA Repair.

[19] Hagenkort A (2017). dUTPase inhibition augments replication defects of 5-fluorouracil. Oncotarget.

[20] Jones CW, Priest DG (1978). Interaction of pyridoxal 5′-phosphate with aposerine hydroxymethyltransferase. Biochim. Biophys. Acta..

[21] Perry C, Yu S, Chen J, Matharu KS, Stover PJ (2007). Effect of vitamin B6 availability on serine hydroxymethyltransferase in MCF-7 cells. Arch. Biochem. Biophys..

[22] Schirch LV, Edmiston M, Chen MS (1973). Serine transhydroxymethylase: Subunit structure and the involvement of sulfhydryl groups in the activity of the enzyme. J. Biol. Chem..

[23] Giardina G (2015). How pyridoxal 5′-phosphate differentially regulates human cytosolic and mitochondrial serine hydroxymethyltransferase oligomeric state. FEBS J..

[24] Zempleni J, Kübler W (1994). The utilization of intravenously infused pyridoxine in humans. Clin. Chim. Acta.

[25] Ueland PM, Ulvik A, Rios-Ávila L, Midttun Ø, Gregory JF (2015). Direct and functional biomarkers of vitamin B6 status. Annu. Rev. Nutr..

[26] Martínez M, Cuskelly GJ, Williamson J, Toth JP, Gregory JF (2000). Vitamin B-6 deficiency in rats reduces hepatic serine hydroxymethyl transferase and cystathionine β-synthase activities and rates of in vivo protein turnover, homocysteine remethylation and transsulfuration. J. Nutr..

[27] Machover D (2018). Enhancement of 5-fluorouracil cytotoxicity by pyridoxal 5′-phosphate and folinic acid in tandem. J. Pharmacol. Exp. Ther..

[28] Schaumburg H (1983). Sensory neuropathy from pyridoxine abuse. A new megavitamin syndrome. N. Engl. J. Med..

[29] Rimland B, Callaway E, Dreyfus P (1978). The effect of high doses of vitamin B6 on autistic children: A double blind crossover study. Am. J. Psychiatry.

[30] Conroy T (2011). For the Groupe Tumeurs Digestives of Unicancer and the PRODIGE Intergroup. FOLFIRINOX versus gemcitabine for metastatic pancreatic cancer. N. Engl. J. Med..

[31] Machover D (1982). Fluorouracil combined with the pure (6S)-stereoisomer of folinic acid in high doses for treatment of patients with advanced colorectal carcinoma: A phase I–II study. J. Natl. Cancer Inst..

[32] Meluch AA (2003). Preoperative therapy with concurrent paclitaxel/carboplatin/infusional 5-FU and radiation therapy in locoregional esophageal cancer: Final results of a Minnie Pearl Cancer Research Network phase II trial. Cancer J..

[33] Schneider M (1995). Phase II trial of cisplatin, fluorouracil, and pure folinic acid for locally advanced head and neck cancer: A pharmacokinetic and clinical survey. J. Clin. Oncol..

[34] Freireich EJ, Gehan EA, Rall DP, Schmidt LH, Skipper HE (1966). Quantitative comparison of toxicity of anticancer agents in mouse, rat, hamster, dog, monkey, and man. Cancer Chemother. Rep..

[35] Machover D (1996). Two consecutive phase II studies of oxaliplatin (l-OHP) for treatment of patients with advanced colorectal carcinoma who were resistant to previous treatment with fluoropyrimidines. Ann. Oncol..

[36] Bachet JB (2018). For FFCD investigators. FOLFIRINOX as induction treatment in rectal cancer patients with synchronous metastases: Results of the FFCD 1102 phase II trial. Eur. J. Cancer..

[37] Ychou M (2013). A randomized phase II trial of three intensified chemotherapy regimens in first-line treatment of colorectal cancer patients with initially unresectable or not optimally resectable liver metastases. The METHEP trial. Ann. Surg. Oncol..

[38] Taïeb J (2018). Exploratory analyses assessing the impact of early tumour shrinkage and depth of response on survival outcomes in patients with RAS wild-type metastatic colorectal cancer receiving treatment in three randomised panitumumab trials. J. Cancer Res. Clin. Oncol..

[39] Heinemann V (2015). Early tumour shrinkage (ETS) and depth of response (DpR) in the treatment of patients with metastatic colorectal cancer (mCRC). Eur. J. Cancer.

